# In Vitro Evaluation of Cellular Interactions with Nanostructured Spheres of Alginate and Zinc-Substituted Carbonated Hydroxyapatite

**DOI:** 10.3390/ma17164092

**Published:** 2024-08-17

**Authors:** Jessica Dornelas, Gisele Dornelas, Elena Mavropoulos Oliveira Tude, Carlos Fernando Mourão, Alexandre da Malta Rossi, Gutemberg Gomes Alves

**Affiliations:** 1 NanoOnco3D, Rio de Janeiro 24033-000, Brazil; 2 Cell and Molecular Biology Department, Institute of Biology, Fluminense Federal University, Niterói 24220-900, Brazil; 3 Post-Graduation Program in Sciences & Biotechnology, Institute of Biology, Fluminense Federal University, Niterói 24220-900, Brazil; 4 CBPF–Brazilian Center for Research in Physics, Rio de Janeiro 22290-180, Brazil; 5 Department of Periodontology, Tufts University School of Dental Medicine, Boston, MA 02111, USA

**Keywords:** hydroxyapatite, zinc, osteoblasts, nanostructured biomaterials

## Abstract

The increasing demand for effective bone regeneration materials drives the exploration of biomaterials with enhanced bioactivity and biocompatibility, such as zinc-substituted compounds. This study investigates the in vitro cellular interactions with nanostructured spheres composed of alginate/carbonated hydroxyapatite (CHA), compared to zinc-substituted CHA (ZnCHA). This work aimed to compare the physicochemical properties and biological effects of ZnCHA and CHA on osteoblasts. ZnCHA was synthesized using a wet chemical method, followed by characterization through X-ray diffraction, Fourier transform infrared spectroscopy, total organic carbon analysis, Wavelength-dispersive X-ray spectroscopy, and BET surface area analysis to assess ion release and structural changes. Biological evaluation was conducted using cell viability, proliferation, and biomineralization assays on osteoblasts. Results showed successful incorporation of zinc and carbonate, leading to reduced crystallinity and increased surface area. Cell viability and proliferation assays indicated ZnCHA’s cytocompatibility and enhanced osteoblastic activity, with increased mineralization nodules compared to CHA samples. The study concludes that ZnCHA composites are promising candidates for bone tissue engineering, demonstrating improved cytocompatibility and potential for further preclinical evaluations.

## 1. Introduction

Bone substitutes are widely investigated due to their market leadership in biomaterials and to the growing demand associated with an aging population. Calcium phosphates (CaP) are the most used synthetic materials for filling bone defects and smoothing contour irregularities on orthopedic, craniofacial, and orthodontic surgeries [1]. Among CaPs, hydroxyapatite (HA, Ca_10_(PO_4_)_6_(OH)_2_) is notable for its high in vitro biocompatibility and in vivo bioactivity [2]. This material produces a bioactive apatite layer, favoring the implant-tissue connection and ensuring an interface with strength comparable to bone [3]. However, as a well-known limitation, hydroxyapatite exhibits low solubility, which limits its resorption rate compared to other materials such as TCP, brushite, and polymeric scaffolds [4,5]. While this characteristic may be interesting to resist removal from bone attachments, especially when associated with bioglass [6], it may limit the substitution of the grafted material by regenerated tissue, which is an important step in bioengineering applications.

Aiming to enhance the performance of HA, studies have shown that the addition of carbonate to the HA structure decreases its crystallinity and increases its solubility, thereby improving osseointegration and new bone formation [7]. Compared to crystalline stoichiometric HA, carbonated hydroxyapatite offers several advantages, including enhanced solubility, improved bioactivity and osseointegration, a composition closer to natural bone, and tunable properties for customized degradation rates to meet specific bone regeneration needs [7]. Furthermore, researchers have also explored incorporating it into composite spheres with alginate, a natural biopolymer known for its biodegradability and ability to encapsulate materials [8]. These CHA-alginate spheres are being optimized for bone regeneration applications as scaffolds in tissue engineering. The controlled degradation of these spheres influences the release of beneficial ions from the CHA core, which can be tailored to match the rate of new bone formation, potentially enhancing osseointegration [9]. Moreover, this composite has the potential for the incorporation of bioactive ions that could contribute to desired biological responses from bone cells.

Zinc (Zn) is a trace element essential for the growth and development of animals and humans. Many studies have reported the biological effects of the incorporation of zinc in materials for bone therapy. Several studies correlated the release of Zn in the culture medium with increased cell proliferation and differentiation of osteoblasts [10]. Zinc is known for its role in enhancing bone metabolism, cell proliferation, and tissue regeneration, with several studies proposing the incorporation of zinc into hydroxyapatite to produce biomaterials that stimulate and accelerate bone healing, as discussed in a recent review [11]. Given the potential therapeutic benefits, substituting carbonate apatite with zinc during synthesis could be advantageous in producing moldable ZnCHA/alginate composite spheres for bone treatment.

To understand the impact of these modifications, it is necessary to investigate how the simultaneous incorporation of Zn^2+^ and CO_3_^2−^ ions affects the release profile of ions from zinc-substituted carbonate apatite (ZnCHA) compared to traditional carbonate apatite (CHA). Additionally, it is important to examine how this incorporation influences the crystal structure of the material and the biological response of bone cells. In this context, this work aimed to (i) evaluate the physicochemical changes resulting from zinc incorporation into the biomaterial structure, (ii) assess the release kinetics of zinc from the material into the culture medium, and (iii) evaluate the biological effects of zinc released by the material on rat femur osteoblastic cells (Fost), focusing on cytocompatibility, cell proliferation, and in vitro calcium phosphate deposition (mineralization) by these cells.

## 2. Materials and Methods

### 2.1. Synthesis and Characterization of the Carbonate Hydroxyapatite Substitute with Zinc

Carbonated hydroxyapatite (CHA) and carbonated hydroxyapatite substituted with zinc (ZnCHA) (Table 1) were produced and characterized in the Bioceramic Laboratory of the Brazilian Center for Physics Research—CBPF (Rio de Janeiro). All reagents were obtained from Merck (Rahway, NJ, USA).

Synthetic ZnCHA was synthesized by a wet method by the addition of a mixture of NH_4_ HPO_4_ (0.11 mol/L) and NH_4_ CO_3_ (0.0012 mol/L) solution dropped (30 mL/min) on a mixture of Zn(NO_3_)_2_ (Merck, Rahway, NJ, USA) and Ca(NO_3_ )_2_ solution at 90 °C and at pH = 13, adjusted by adding (0.5 mL/min) an aqueous solution of NH_4_OH (14 mol/L). The precipitates were aged for 2 h, filtered, and washed three times with Milli-Q water. The dried powder was manually macerated, and particles with particle sizes below 74 μm were separated using 200, 110, and 74 μm precision sieves (Bertel Metalurgica Ltd., Sao Paulo, Brazil). For the synthesis of the control material, carbonated hydroxyapatite (CHA), the same route was used, excluding the reagents Zn(NO_3_)_2_ and NH_4_CO_3_ for the synthesis of CHA.

### 2.2. Alginate-Apatite Microspheres Processing

In order to prepare the spheres, a mixture of each sample (CHA or ZnCHA) containing 1% alginate was prepared by slowly adding 1 g of sodium alginate into 10 mL of ultrapure water (Milli-Q) (Fluka, Sigma-Aldrich, St Louis, MO, USA) at a ratio of 1:15 (0.1 g of alginate to 1.5 g of biomaterial) under constant magnetic stirring. Each of these biomaterial suspensions was extruded with a speed rate of approximately 10 mL/h with a 0.7-mm diameter needle in 0.3 M CaCl_2_ (Vetec—Rio de Janeiro, Brazil) and left to ion exchange for 24 h. Afterward, the formed beads were washed with ultrapure water (Milli-Q) until the pH was equal to 7 and dried at room temperature. The spheres were sieved in the range of >600–<850 μm using a precision sieve (Bertel Metalurgica Ltd., Sao Paulo, Brazil).

### 2.3. Sample Characterization

Powders were characterized by X-ray diffraction (XRD, HZG4, Zeiss, Oberkochen, Germany) using a diffractometer operating with CuKα radiation (1.5418 Å) at 40 kV and 40 mA with a graphite monochromator in the primary bunch. The XRD patterns were obtained in 2θ step intervals from 10 to 100°.

Fourier transform infrared (FTIR) spectroscopy (Shimadzu IR-Prestige-21/AIM-880, Kyoto, Japan) analyses of powders were performed using KBr pellets, and spectra with 20 scans in the wavenumbers range of 4000 and 400 cm ^−1^ were recorded in the transmission mode, diluted in potassium bromide. The reading was normalized with the 3-point baseline correction using the IR Prestige-21 software (Lab Solutions IR version 2.3).

The Ca/P molar ratio was calculated from the data measured by Wavelength-dispersive X-ray spectroscopy (WDS) using a PW 2400 Sequential Wavelength X-ray Spectrometer (Philips, Cambridge, MA, USA) at 3.0 KV. Samples were prepared by fusion with lithium tetraborate (Li_2_B_4_O_7_), using calcium oxide (CaO), sodium phosphate (Na_3_PO_4_), and zinc oxide (ZnO) as elemental standards for the detection of Ca, P, and Zn.

The carbonate content was measured by total organic carbon (TOC) analysis using a SC-144DR Sulfur and Carbon Analyzer (LECO Corp., Hackettstown, NJ, USA). The samples were powdered and taken to an oven in an atmosphere of super dry oxygen at a temperature of 1350 °C, where they underwent an oxy-reduction process and the carbon present in the sample was converted to CO_2_.

Surface area (BET) of all powders was determined using a physisorption technique by the ASAP 2020 Accelerated Surface Area and Porosimetry Analyzer (Micrometrics, Norcross, GA, USA). For the measurements, the samples were cooled to a temperature of −195.4 °C, previously immersed in liquid nitrogen, and maintained at the same temperature during the analyses.

The structure of the CHA and ZnCHA spheres was analyzed by scanning electron microscopy (SEM) using a JSM-7100F electron microscope (JEOL, Tokyo, Japan). For the internal evaluation of the sphere organization, cross-sections of different thickness were made using an ultramicrotomy blade with a Leica EM UC7 ultramicrotome (Leica, Wetzlar, Germany). 

### 2.4. Dissolution Behavior in Cultural Medium

To evaluate the material dissolution behavior, alginate-apatite microsphere extracts were obtained after 14 days of incubation with D-MEM High culture medium (Life Technologies, Carlsbad, CA, USA) without FBS. The extracts from each sample were kept under orbital shaking at room temperature, and at the end of each day, the supernatant of the extracts was collected and replaced with D-MEM High culture medium without FBS. The extracts were filtered through a 0.22 µm Durapore membrane (Millipore, Burlington, MA, USA). Ca and Zn concentrations were determined by Inductively Coupled Plasma (ICP) using an Optima 3000 DV ICP System (Perkin-Elmer, Waltham, MA, USA).

### 2.5. Cell Viability 

Murine osteoblasts (F-Ost) isolated from mice (Balb-c) were obtained from the collection at the National Institute of Trauma and Orthopedics (INTO). These cells were previously characterized as reported elsewhere [12]. For the cytocompatibility evaluation, the murine osteoblast cell line FOst (#8-16) was cultured in Dulbecco’s Modified Eagle Medium high glucose (D-MEM high glucose) supplemented with 10% fetal bovine serum (FBS) (Life Technologies, Auckland, New Zealand) and incubated at 37 °C in a 5% CO_2_ atmosphere. Confluent low passages were trypsinized, counted using a Neubauer chamber, and used in all experiments. The control group for each assay was seeded on plates with DMEM High Glucose supplemented medium. Sample extracts were collected for the cell viability assay after the centrifugation procedure described above. A 1% SDS (sodium dodecyl sulphate) (Sigma, USA) solution was used as the positive cytotoxicity control and polystyrene pellets (100 mg/mL) as the negative cytotoxicity control. FOst osteoblasts were seeded in a 96-well cell culture plate (0.05 × 10^4^ cells/well) and cultured in D-MEM supplemented with 10% FBS for 24 h at 37 °C in a 5% CO_2_ 95% atmosphere. After 24 h of cell exposure to the extract media, cytotoxicity was evaluated with a Prestoblue assay reagent (Invitrogen, Waltham, MA, USA). Proliferation was monitored at 3, 7, 10, and 14 days by reading Prestoblue, at the end of each fresh extract. The colorimetric assays were performed with the help of a Synergy II UV/Vis spectrophotometer (Biotek Instruments, Winooski, VT, USA). On the seventh day of the experiment, each extract was replaced by a fresh extract every 72 h.

Cells exposed to CHA and ZnCHA-conditioned media were also evaluated to identify possible morphological changes induced by nanoparticles. Cells were plated on glass coverslips and, after exposure to conditioned medium, fixed with Karnovsky’s solution and washed with cacodylate buffer. The samples were then treated with osmium tetroxide (1:1) (Sigma-Aldrich, St. Louis, MO, USA) and dehydrated through a series of increasing concentration alcohol baths (30–100%), completing dehydration with HMDS (Sigma-Aldrich, St. Louis, MO, USA). The samples were gold-coated using a Denton Vacuum Desk V and observed at 10 kV with a magnification of 1500× using secondary electron detection on a scanning electron microscope (SEM) model JSM-7100F (JEOL).

### 2.6. Mineralization Assay

To test if the materials are able to induce in vitro mineralization of osteoblasts in osteogenic conditions, osteoblasts (FOst) were seeded at 50,000 cells/cm^2^ in 100 mm tissue culture dishes and allowed to adhere for 24 h at 37 °C/5% CO_2_ in a humidified incubator. Cells were exposed to conditioned medium (CHA and ZnCHA) from the spheres (at a ratio of 100 mg/mL) supplemented with medium supplemented with β-glycerophosphate at different concentrations of 1, 2.5, 5, 7.5, and 10 mM (Sigma, USA). The cell cultures were maintained for 14 days, with the culture medium being replaced every 3 days.

Mineralization of the cultures was visualized directly in the culture dishes using 40 mM of Alizarin Red-S (Sigma-Aldrich, St. Louis, MO, USA) and observed with an inverted light microscope (Axio A1, Zeiss, Oberkochen, Germany). Quantitative analysis was performed by extracting the Alizarin Red-S dye with 10% acetic acid (Sigma-Aldrich, St. Louis, MO, USA), added to each well for 30 min, then the cells were gently scraped and the entire contents centrifuged, recognizing only the supernatant, which was adjusted to pH 4.1–4.5. Quantification of the released dye was performed using a spectrophotometer at 405 nm (Sinergy II, Biotek Instruments, USA).

### 2.7. Immunofluorescence Assay

The presence of RUNX-2 was monitored by immunostaining. Mature osteoblasts (F-Ost) were seeded at a density of 0.2 × 10^4^ in a 24-well plate. After 7 days, the cells were fixed with 4% PFA (Sigma-Aldrich, St. Louis, MO, USA) and processed for optical fluorescence microscopy, where 2 μg/mL of primary antibodies to RUNX-2 (SC-390351, Santa Cruz Biotechnology, Dallas, TX, USA) were used, with the cell nucleus being labeled with DAPI (4 ‘6-diamidino-2-phenylindole Fluoroshield, Sigma-Aldrich, St. Louis, MO, USA). The samples were analyzed using an optical fluorescence microscope (Axio Observer A1, Zeiss, Germany). Using the Image J software (v1.54f), the fluorescence intensity was quantitatively analyzed to estimate the production of the analyzed markers.

### 2.8. Statistical Analysis

For the dissolution tests, the statistical analysis used was the T-student test. For cell viability tests, after determining normality using the D’Agostino Pearsons test, the one-way ANOVA test and Dunnett’s post-test were used, which compares each sample with the negative control. All tests were performed using the GraphPad Prisma 8 software (8.0.1), using a 95% confidence interval (*p* < 0.05).

## 3. Results

Chemical analysis by Atomic Absorption and TOC revealed the incorporation of carbonate in the CHA and ZnCHA powders, with respective values of 2.4% and 3.3% (Table 2). The zinc content in the ZnCHA sample was approximately 3% Zn, lower than the expected 5% content for the preparation. However, this lower zinc content seems to be compensated by the lower charge of carbonate substitution (CO_3_^−2^) on anionic phosphate (PO_4_^−3^) sites, and the resulting (Ca + Zn)/P ratio remains close to apatite stoichiometry.

**Figure 1:**
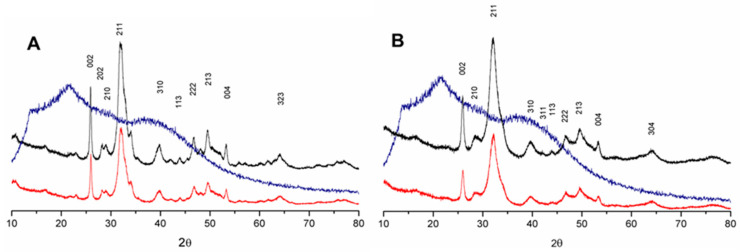
XRD pattern diffractograms of the samples of CHA (**A**) and ZnCHA (**B**). The samples’ power and biocomposites show characteristic peaks of calcium phosphate. Alginate is represented by the blue line. CHA and ZnCHA power are represented by a red line. Biocomposites CHA + Alginate and ZnCHA + Alginate are represented by the black line.

Although the ionic co-substitution induces a decrease in the sample Ca/P ratio, the calcium phosphate crystalline phase remained the same. The XRD parameters, represented in Figure 1, show only one phase in CHA and ZnCHA powders. The indexed peaks 002, 210, 211, 202, and 310 were related to parameters determined by the JCDPS (n° 09-0432) characteristics of hydroxyapatite.

**Figure 2:**
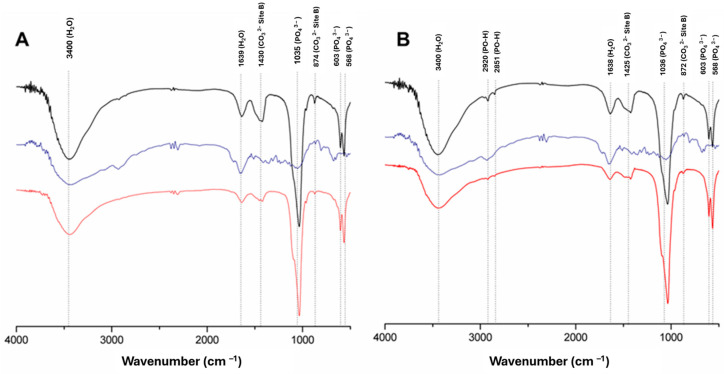
Fourier transform infrared (FTIR) spectra of CHA (**A**) and ZnCHA (**B**). The samples’ power and biocomposites show characteristic bands of calcium phosphate. Alginate is represented by the blue line. CHA and ZnCHA power are represented by a red line. Biocomposites CHA + Alginate and ZnCHA + Alginate are represented by the black line.

In the FTIR spectra of the materials (Figure 2), analyzing the position of the peaks 002 and 300 and based on the Sherrer equation, the estimated crystallite size of the samples was determined, as shown in Table 1. The value of the ratio between plans 002/300 in the CHA was 2.1 and in the ZnCHA of 1.8, respectively. Among the samples, there was no statistical difference regarding crystallite size, indicating that co-substitution in the ZnCHA sample did not alter the crystalline form compared to CHA (Figure 2).

**Figure 3:**
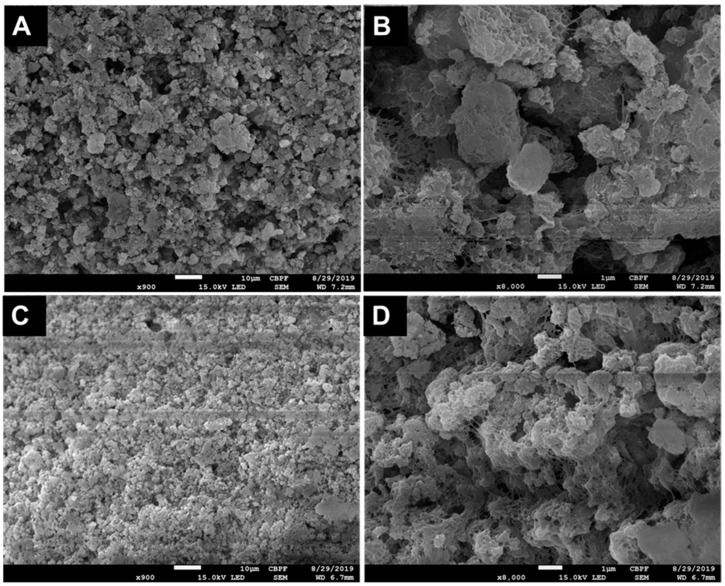
Cross-sectional SEM microphotographs of the CHA and ZnCHA microspheres show sample microstructure and grain distribution. (**A**: CHA at 900× magnification; **B**: CHA at 8000×; **C**: ZnCHA at 900×; **D**: ZnCHA at 8000×).

The surface of CHA and ZnCHA microspheres was analyzed by BET to determine specific area, mean pore size, and pore volume, as shown in Table 3. ZnCHA microspheres present a higher specific area and pore volume due to smaller crystal dimensions. Morphological analysis of the spheres by SEM (Figure 3) shows the distribution of grains in the structure of the spheres. In the CHA sample, there are greater interceptions between grains when compared to ZnCHA samples. The surface of the ZnCHA spheres appears more uniform in the organization of the grains, forming microchannels inside the material.

**Figure 4:**
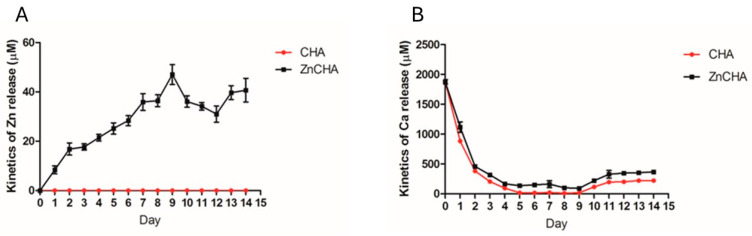
Ion release/precipitation kinetics of CHA and ZnCHA microspheres on D-MEM high glucose culture medium analyzed over 14 days: (**A**) Dynamics of zinc ion release/precipitation and (**B**) Dynamics of calcium ion release/precipitation.

The kinetics of ion release of the CHA and ZnCHA microspheres on D-MEM high glucose culture medium were evaluated by Atomic Absorption (AA), as shown in Figure 4. Calcium and zinc concentrations were monitored over the 14-day period. In the CHA and ZnCHA samples, calcium levels were strongly reduced throughout the period of exposure of the material to the culture medium, revealing the calcium sequestration and/or deposition from the medium to the material. While the calcium concentrations of the medium decreased, zinc concentrations increased, showing that the material released zinc incorporated into the ZnCHA sample, reaching 40 μM of zinc released after 8 days.

**Figure 5:**
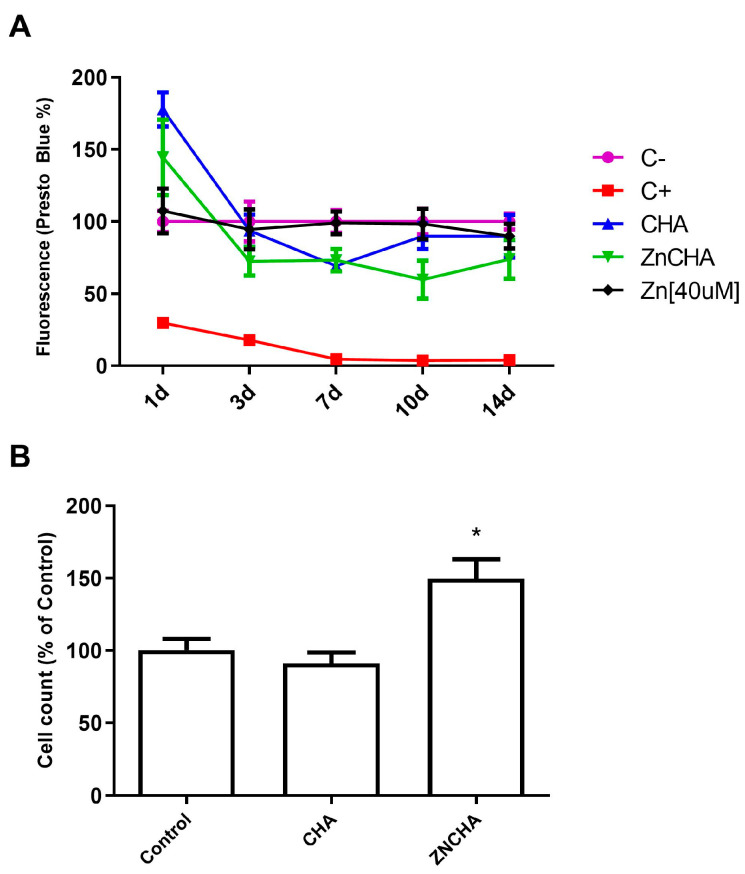
Cytocompatibility by indirect contact in the 14-day period. Negative control (C−) was cultured in a supplemented culture medium, and positive control was supplemented with 1% SDS and extracted from the respective CHA and ZnCHA microspheres samples supplemented with 10% FBS. The cell viability was monitored by Presto Blue (**A**) and cell proliferation at 7 days by cell counting (DAPI staining and fluorescence microscopy). (**B**). An asterisk (*) indicates a statistical difference between groups (*p* < 0.05).

The cytocompatibility test using FOst osteoblasts showed that both the CHA and ZnCHA groups were cytocompatible over prolonged exposure periods (14 days), with similar cell levels when compared to the experimental control group (cells exposed to culture medium) by Presto Blue reagent (Figure 5A). Furthermore, the results observed in the CHA and ZnCHA microsphere samples at the 7th day after exposure were higher than the negative control (polystyrene) in terms of cell density, indicating high biocompatibility of the biocomposites (Figure 5B).

**Figure 6:**
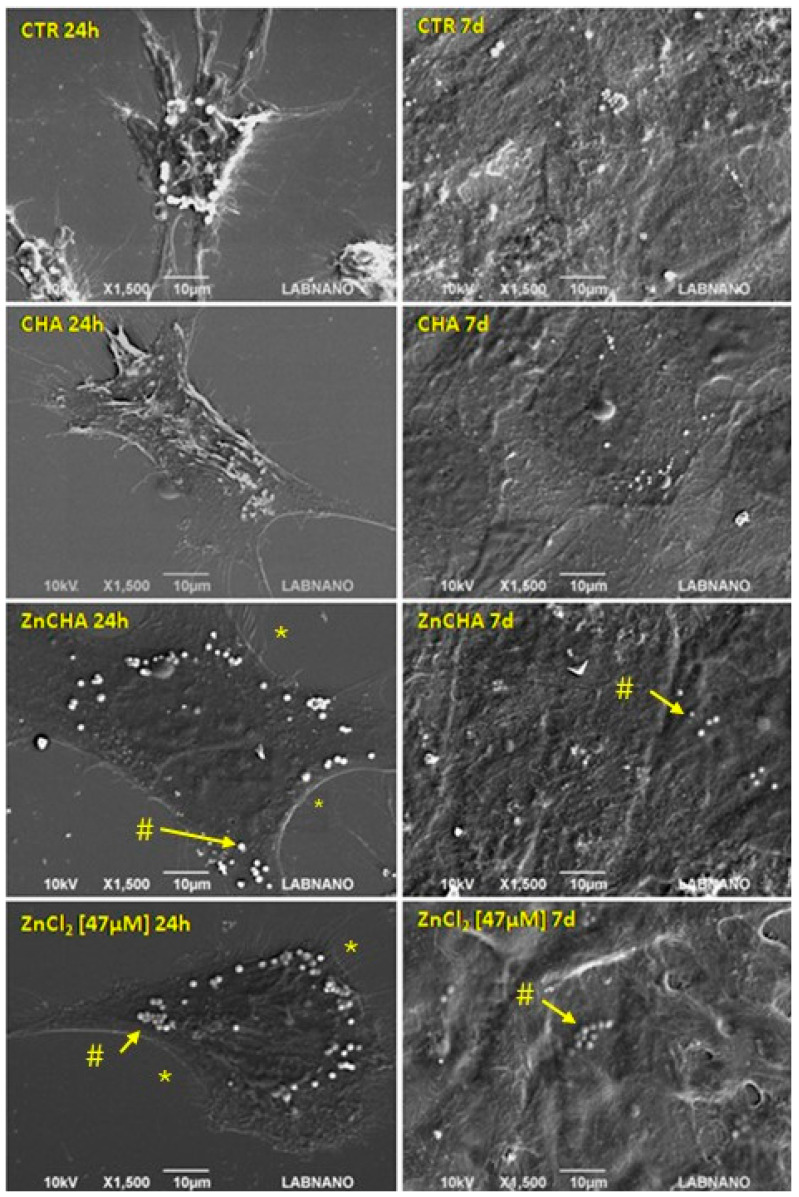
Morphological analysis of the interaction of F-Ost cells (osteoblasts) with conditioned medium and analyzed at 24 h and 7 d. Cells were exposed to the supplemented medium (control, CTR), CHA and ZnCHA-conditioned media, or 47 μM ZnCl_2_. Within 24 h, the presence of filopodia (*) and vesicular (#) formation on the cell surface, with the exception of CHA. In 7 days, complete coverage of the glass coverslip is observed, with cellular stratification.

Knowing that the medium underwent changes in terms of ionic concentrations and the presence of biocomposite aggregates (CHA and ZnCHA), the possible interference of these on cell morphology was investigated. Cells were analyzed by SEM on days 1 and 7 (Figure 6). It can be observed that in all groups, the F-Ost cells exhibited the characteristic morphology of osteoblastic cells, presenting filopodia and lamellipodia. However, in the ZnCHA and 47 μM Zn samples, the presence of vesicles on the surface of the cells was observed, indicative of cellular activity in collagen production—a feature not noticed in the cells in culture medium and CHA on days 1 and 7. On the 7th day, in all groups, there was the formation of a stratified layer of cells, indicating high cell adhesion and no interference with cell viability. 

**Figure 7:**
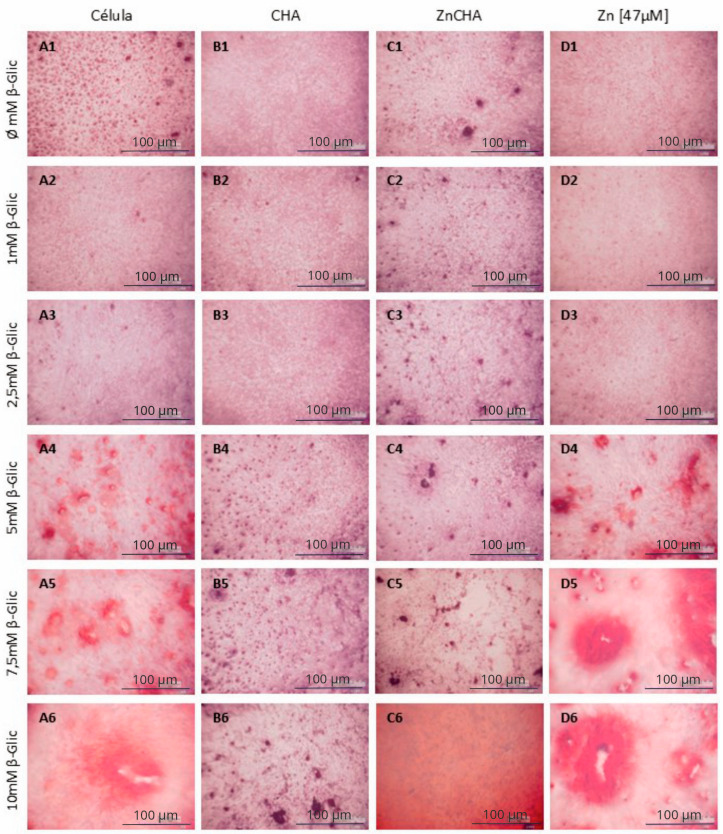
Analysis of biomineralization of osteoblastic cells FOst in contact with CHA and ZnCHA extract as a function of the variation of β-glycerophosphate concentration. Cell Control (**A1**–**A6**), CHA Extract (**B1**–**B6**), ZnCHA Extract (**C1**–**C6**), and ZnCl_2_ Solution [47 μM] (**D1**–**D6**). The cells show the formation of mineralization nodules in the groups: Cell, ZnCHA, and ZnCl2 [47 μM]. Only from the 5 mM concentration of β-Glycerophosphate, the cells start responding to the stimulus to initiate the mineralization process.

**Figure 8:**
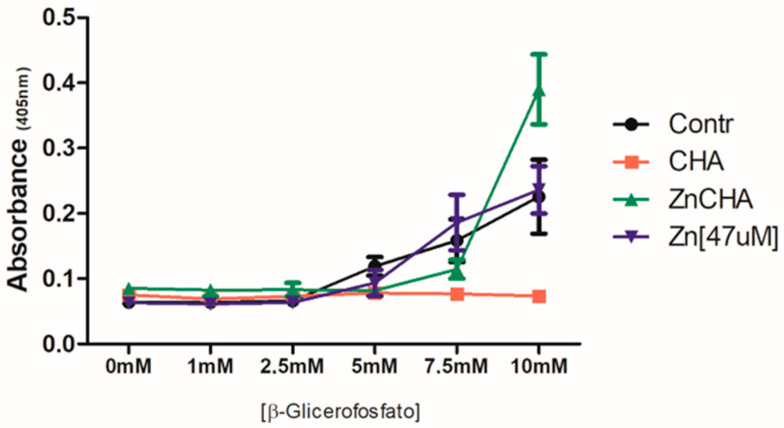
Quantitative analysis of the biomineralization through the extraction of Alizarin Redand absorbance at 450 nm from the nodules of biomineralization. Evaluation of the formation of mineralization nodules as a function of the variation of β-Glycerophosphate concentration in the groups: Cells (black line), CHA (red line), ZnCHA (green line), and ZnCl_2_ [47 μM] (blue line). From the concentration of 5 mM of β-Glycerophosphate the groups of Cells, ZnCHA, and 47 μM ZnCl_2_ presented the formation of mineralization nodules and were increased in higher concentrations of β-Glycerophosphate. ZnCHA presented a statistical difference compared to the control at a 10 mM concentration of β-Glycerophosphate (*p* < 0.05).

The biomineralization assay evaluated the exposure of FOst osteoblasts to the conditioned medium to the CHA and ZnCHA spheres on the variation of β-glycerophosphate concentrations, with the mineralization nodules revealed by Alizarin Red-S. Osteoblastic cells initiated the formation of mineralization nodules from the stimulus with 5 mM β-Glycerolphosphate (Figure 7). The group of cells exposed to the CHA-conditioned medium did not present mineralization nodules as observed in control. The groups exposed to the conditioned medium with ZnCHA and 47 μM ZnCl_2_ presented the formation of mineralization nodules. The group treated with ZnCl_2_ showed a similar response to the control, suggesting that the presence of Zn^2+^ present in the medium was not enough to promote the increase in the formation of mineralized nodules. However, the group exposed to the conditioned medium with ZnCHA associated with a higher concentration of β-Glycerolphophate (10 mM) showed a significant increase in the formation of nodules when compared to the control, showing that the presence of ZnCHA particles present in the culture medium promoted an increase in the induction of osteoblastic cells for the formation of mineralization nodules (Figure 8).

**Figure 9:**
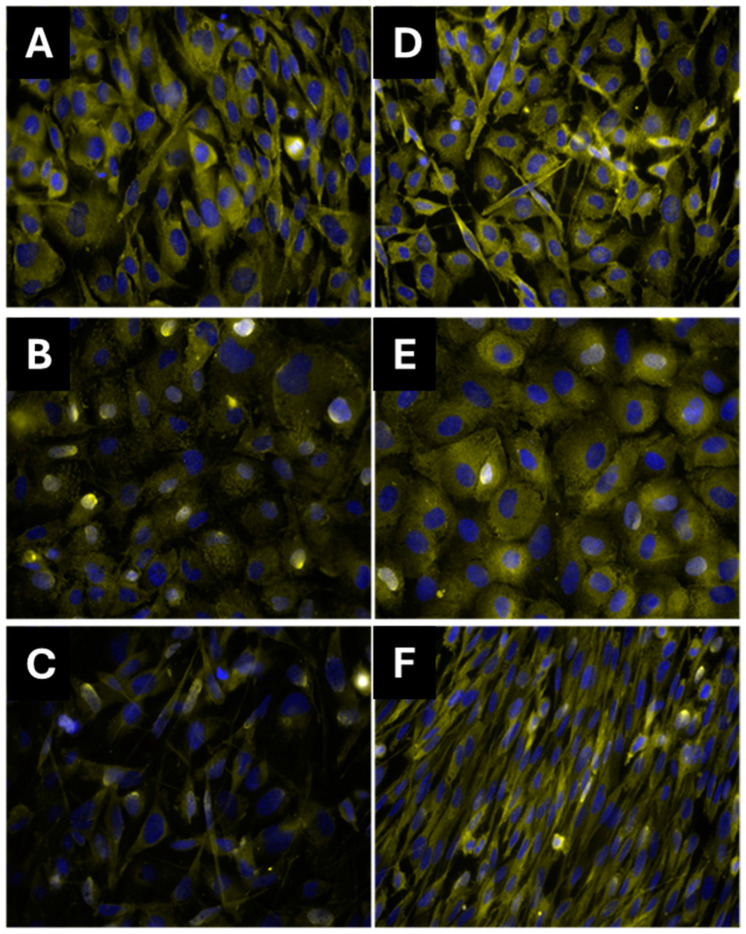
Immunostaining of osteoblasts after indirect exposure to CHA and ZnCHA biocomposite extracts for 7 days. Core in (blue) and Runx-2 (yellow). Control (**A**,**D**); CHA (**B**,**E**); ZnCHA (**C**,**F**). Samples (**D**–**F**) were induced with 10 mM β-glycerophosphate to stimulate cellular osteogenic activities.

**Figure 10:**
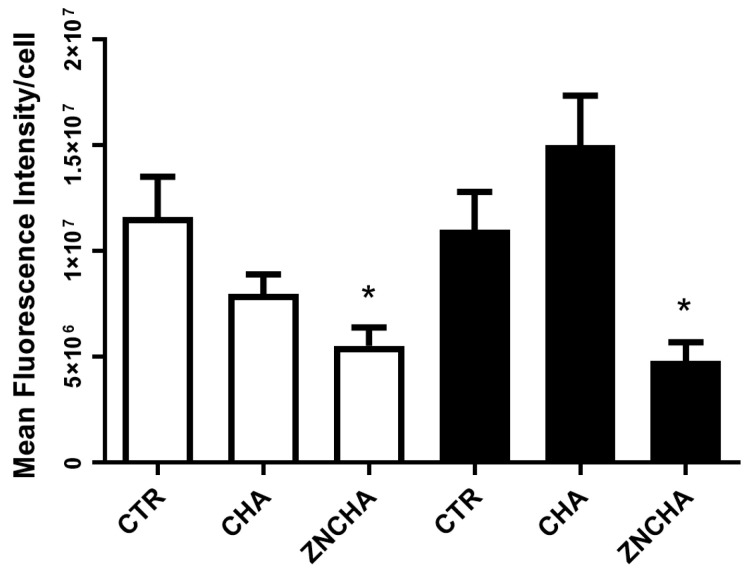
Quantification of Runx-2 immunostaining by number of cells after exposure to CHA, ZnCHA, and ZnCl2 [47 uM] extracts for 7 days, samples without β-Glic □ and with β-Glic ■. ZnCHA with and without induction, marked with an asterisk (*), showed a significant difference in relation to the control (cells) (*p* < 0.05).

An analysis was carried out based on the observation of labeled Runx-2 (Figure 9), which was detected by immunofluorescence over a period of 7 days in the presence and absence of β-Glyc. Runx-2 was identified in all groups. In the ZnCHA group, an increase in the number of stained cells was observed compared to the other groups analyzed. Fluorescence intensity was quantified, and statistical analysis revealed that the CHA with β-Glyc group showed an apparent increase in the quantification of Runx-2, but it was not significant (*p* > 0.05). The ZnCHA groups with and without β-Glyc showed a significant reduction in fluorescence intensity compared to the control (*p* < 0.05) (Figure 10).

## 4. Discussion

In recent decades, intense research into alloplastic biomaterials has been developed, with nanostructured calcium phosphates considered among the most promising in regenerative medicine due to their chemical and structural similarity to bone [13]. Synthetic hydroxyapatite (HA) is a calcium phosphate (CaP) recommended for use in bone repair due to its good biocompatibility and bioactivity [14]. While its low absorption by the body hinders the osseointegration of the material, low synthesis temperatures and ionic substitutions with carbonate and zinc can make HA less crystalline and more soluble [7]. Furthermore, it is known that zinc enhances biological responses in osteoblasts, accelerating proliferation and increasing the production of bone biomarkers during mineralization [10]. This study investigated the double substitution of carbonate (CO_3_^2−^) and zinc (Zn^2+^) in the structure of hydroxyapatite to produce zinc-substituted carbonatoapatite (ZnCHA), evaluating physicochemical modifications, ion and particle release in culture medium, and the impact on osteoblasts.

During biomaterial development, it is crucial to verify the components and degradation process, ensuring the material is safe and meets the characteristics of a good bone substitute before in vivo testing. Thus, a physicochemical characterization of ZnCHA was carried out to determine variations in crystallinity, chemical composition, and dissolution. The incorporation of CO_3_^2−^ into the HA structure, as reported by Anjos et al. [15], did not cause the formation of a new crystalline phase but reduced crystallinity. Replacing the PO_4_^3−^ group with CO_3_^2−^ deformed the crystallite, changing its dimensions in the ab and c planes [16]. Additionally, low synthesis temperatures contribute to increased crystal deformity, forming smaller crystals. Metal substitutions promote similar deformity, as observed by Lala et al. [17], who tested varying concentrations of Zn doping in HA. Zinc incorporation preferentially replaces the Ca1 site, reducing the 002 plane size and the crystallite size, causing XRD peak displacement, and increasing the amorphous character of the material, favoring dissolution [18]. In this work, similar patterns were observed in the XRD analysis and refined using the Scherrer equation. The crystallite dimensions of the CHA and ZnCHA samples were reduced in the 002 and 300 planes, confirming that ZnCHA corresponds to HA with structural modifications due to the simultaneous incorporation of CO_3_^2−^ and Zn^2+^.

During HA crystal nucleation, different groups characteristic of CaPs are formed, such as PO_4_^3−^, H_2_O, and CO_3_^2−^, detected by corresponding vibrational bands in FTIR. In samples without heat treatment, H_2_O bands are present (3400 cm^−1^ and 1632 cm^−1^) within the crystal structure [19]. In the HA structure, PO_4_^3−^ emits vibrations, forming the bands (v3) 1065 cm^−1^ and (v4) 568 cm^−1^. The presence of CO_3_^2−^ is confirmed by the bands 1458 cm^−1^ and 873 cm^−1^, corresponding to vibrations of CO_3_^2−^ that replaced PO_4_^3−^ [20]. The results indicate the main bands in CHA and ZnCHA even after the simultaneous replacement of CO_3_^2−^ and Zn^2+^.

Chemical analysis by Atomic Absorption and TOC revealed carbonate incorporation in CHA and ZnCHA powders, with respective values of 2.4% and 3.3%, indicating that zinc presence enhances the carbonate content. Similar observations were reported by Scudeler et al. [21] using FTIR and thermal analysis of HA, ZnHA, and SrHA, which attributed this phenomenon to a hydrated surface layer. The CO_3_^2−^ and Zn^2+^ proportions are expected to directly interfere with the chemical composition, as Zn^2+^ incorporation reduces Ca^2+^ in the structure. Due to the atomic radii difference between Ca (180 Å) and Zn (134 Å), Zn replacement in the HA lattice reorganizes the crystal, delaying nucleation and making the sample calcium-deficient [17]. This is observed by the reduced Ca/P ratio compared to stoichiometric HA [22]. Although the theoretical Zn concentration during synthesis was 5%, only 3% was incorporated, while CHA can reach Zn saturation near 15% [17]. 

Previous studies compared the binding energy of these elements when incorporated into HA, estimating the type of chemical bond made in its crystalline structure and identifying the formation of different compounds [23]. In this work, the P2p binding corresponding to PO_4_^3−^ was 133.22 eV in CHA and 132.86 eV in ZnCHA, suggesting HA formation despite the substitutions. Ca2p binding energy in CHA (347.22 eV) was close to Ca(HPO_4_), while ZnCHA’s 346.86 eV was closer to HA structure. Zn2p3/2 binding energy in ZnCHA samples was 1021.86, indicating it is bound to the tetrahedral oxygen site [23]. The binding energy identified for CO_3_^2−^ (284.22 eV and 284.36 eV in CHA and ZnCHA, respectively) was in the range described previously by. Goloshchapov et al. [24]. Altogether, these results confirm CO_3_^2−^ and Zn^2+^ replacement due to high binding energy, indicating incorporation rather than adsorption. Despite substitutions, the main HA elements’ chemical bonds were not altered, as shown by binding energy, strongly indicating CO_3_^2−^ and Zn^2+^ replacement in HA structure.

Composites are commonly used in bone filling, where biomaterial and polymer mixtures build structures adapted to the graft site. This work used alginate as a polymer for the formation of spherical composites. Alginate is widely used in granule production due to its biocompatibility and lack of immunological response when implanted. At low pH levels (pH 3≈3.5), alginate becomes highly viscous, but at biological pH (pH 7.4), viscosity is reduced, allowing nanostructured biomaterial release [25]. This type of composite granules may contribute to surgical bone regeneration as they favor intragranular space formation, allowing biological fluid passage, releasing biologically active elements, and accelerating healing [26,27,28]. Evaluating the composites’ surface, CO_3_^2−^ and Zn^2+^ incorporation promoted changes beyond chemical composition, modifying the materials’ macrostructure. ZnCHA composites showed increased pore volume and surface area compared to CHA. Due to ZnCHA’s smaller crystal size, better compaction with alginate forms a more homogeneous surface. Interconnected pore formation during scaffold production is suitable for nutrient, biological fluid, gas, and cell diffusion [29]. Both materials presented an irregular surface due to agglomerate compaction associated with alginate. Pore diameter distribution in CHA and ZnCHA composites was similar, but ZnCHA samples had more pores in the 0–10 nm range. Surface changes interfered with composite dissolution. CHA quickly lost its sphere structure and adsorbed more Ca between days 5 and 9. ZnCHA composite degradation was slower, with high surface compaction not interfering with Zn release in the aqueous medium, showing material composition and structure contribute to solubility and degradation. 

Immediately after grafting, a biomaterial is surrounded by biological fluids, and blood plasma proteins form a covering around the implant, modifying its surface and dissolution process. Hoppe et al. [30] suggest understanding dissolution is key to developing a material for tissue engineering. To this end, several studies use aqueous solutions varying in chemical composition and pH. In this context, the use of culture medium assesses the dissolution process in a complex solution (with amino acids, vitamins, ions, and other elements), contributing to understanding dissolution effects in vitro and biological impact [31,32]. This study evaluated CHA and ZnCHA composites’ ion adsorption/desorption in D-MEM culture medium. Composite-conditioned medium showed 60% Ca adsorption in the first 24 h, increasing to 95% on the 8th day. CaP has a great affinity for Ca, enabling new apatite (bone-like apatite) deposition. Gustavsson et al. [31] reported constant, gradual Ca ion adsorption from the medium, corroborating D-MEM results due to high Ca concentration in composites, stimulating new apatite deposition.

Given D-MEM medium changes suggesting Ca deposition, the bioactivity of the composites was tested in McCoy medium. Bioactive material Ca deposition is expected to form “bone-like apatite”, a new HA crystalline phase covering the implant, improving interaction with natural bone tissue [33]. Bioactivity tested in McCoy’s medium with biocomposites showed daily Ca concentration reduction, indicating new apatite formation. Interestingly, FTIR analysis of remaining solid materials from CHA and ZnCHA composites indicated a new phase deposition by ZnCHA samples, presenting new bands at 2956 cm^−1^ (CH_3_) and 2924 cm^−1^ (CH_2_). While previous studies show that Zn reduces material bioactivity, hindering crystal nucleation [17], the CH_3_ and CH_2_ band formation identified in this work may relate to a new compound deposition on the HA surface, often related to organic material presence, as observed by León-Mancilla et al. [34] in collagen samples. This suggests ZnCHA samples adsorbed McCoy medium amino acids, a trait that could potentially favor material interactions when implanted in vivo.

Assessing ion release is crucial when investigating Zn-substituted biomaterials, as it directly influences the material’s bioactivity, cellular responses, and overall effectiveness in promoting bone regeneration. The assessment of Zn release from ZnCHA composites in D-MEM high glucose showed gradual Zn^2+^ release over 14 days, with the highest concentration being 47 µM on the 9th day. Lima et al. [32] evaluated ZnHA composites with 0.1% Zn, releasing 1 ppm Zn in 24 h in D-MEM. Biologically active ion release interferes with osteoblast cellular response, as expected for Zn^2+^. While Calasans-Maia et al. [27] observed Zn release from CaP implantation sites, promoting new bone formation, the ideal concentrations of zinc ions for specific responses modulating mineralization still need determination. However, when evaluating the dissolution dynamics of the Ca^2+^ ion, we observed precipitation rather than release, which is due to the properties of CaP, as there is a strong capacity for nucleation of new hydroxyapatite crystals, forming “bone-like apatite”. This dynamic was observed by Anjos et al. [15], who correlated the reduction in calcium concentrations in the culture medium with the high driving force of the medium favoring the nucleation of new HA nanocrystals, as indicated by the reduction in available Ca^2+^ concentrations in the supernatant. Nevertheless, the proliferation at 7 days, identified directly by cell counting, confirms the relevant effect expected for zinc-doped materials on this biological property of osteoblasts [9]. 

Since modified biomaterials must be biocompatible for long-term organism presence, all of them should be tested in vitro before preclinical tests to avoid toxicity and chronic inflammatory responses. The present findings confirmed both CHA and ZnCHA composite cytocompatibility by indirect exposure of bone cells to conditioned medium for up to 14 days. The high cell viability observed with the Prestoblue assay at 24 h possibly reflects increased metabolic activity in response to cell growth and filling the culture well space, along with a high availability of nutrients. This initial effect disappears after 3 days, where a reduction in metabolic conversion rates is observed, represented by the reduction in resazurin conversion, possibly related to the increase in cell density. Despite observed changes in culture media, cells showed no cytotoxic responses to the materials. Furthermore, all cells showed similar morphology to the control at 24 h. After 7 days, material agglomerates close to cells, filopodia, and membrane projections were observed in all groups, possibly responding to NPs presence, and vesicles possibly suggesting mineralization beginning were observed in ZnCHA and 47 μM Zn samples. Osteoblasts are known for their increased production of matrix vesicles during the mineralization phase of osteogenesis. These vesicles, rich in calcium, phosphate ions, and enzymes like alkaline phosphatase, serve as nucleation sites for hydroxyapatite crystals, the main mineral component of bone. The vesicles facilitate the initial mineralization process within the collagen matrix, which is crucial for forming mature bone tissue. The production and release of these vesicles are tightly regulated, ensuring proper bone formation and remodeling [35]. Other studies evaluate Zn-substituted HA cytocompatibility. Lima et al. [32] tested indirect contact impact on cell viability from medium changes with 1 ppm Zn release, noting increased cell viability within 24 h in ZnCHA samples. In a general manner, the cytotoxicity of zinc-containing HA will be relevant at incorporation above 8% [36].

Many studies seek the development of novel smart biomaterials favoring new bone tissue formation, as usually CaP-based materials are not osteoinductive, i.e., unable to induce cells to produce bone matrix. Before matrix mineralization, bone growth factors released by osteoblastic cells modulate the process. Runx2 (Runt-related transcription factor 2) is a critical transcription factor for early osteoblast differentiation, promoting maturation and commitment to bone matrix formation [37]. In osteoblasts, this gene modulates factors including alkaline phosphatase (ALP), osteocalcin (OCN), osteopontin (OP), and type I collagen (Col-I). Its expression is upregulated in preosteoblasts and reaches its maximum level in immature osteoblasts. However, as osteoblasts mature, Runx2 expression is downregulated [38]. This downregulation is essential as it marks the transition from proliferation to the maturation phase, where osteoblasts begin to produce and mineralize bone matrix. Interestingly, Zn acts as a co-factor in many bone metabolism enzymes, and studies show that Zn addition to HA alters Runx-2 expression in osteoblasts [39]. In the present work, all groups showed Runx-2 labeling in F-Ost cells (mature osteoblasts), but only CHA treated with osteoinductive medium showed increased labeling, while ZnCHA samples significantly reduced Runx2 staining compared to control. Runx-2 expression reduction may relate to cell maturity level, where mature osteoblasts reduce Runx-2 levels, suggesting cells entering mineralized matrix production phase [37]. However, more detailed tests are needed to prove ZnCHA’s relationship with bone marker expression modulating mineralization nodule formation in vitro.

As mineralization occurs in response to osteogenic marker expression, osteoblasts deposit organic matrix undergoing CaP nucleation, forming mineralization nodules. A systematic review of animal studies with zinc-doped CaP biomaterials [40] reported that zinc incorporation did not affect its biocompatibility, tends to reduce the resorption rates, and increases osteoconductivy and new bone formation. The present study induced matrix mineralization by the addition of either β-Glycerolphosphate (β-Glyc), a well-known osteogenic inductor, and the presence of media conditioned with either CHA or ZnCHA composites. The ZnCHA + β-Glyc group showed significant mineralization nodule increase, despite low osteogenic marker concentrations, corroborating results observing greater bone formation in ZnCHA-implanted defects [28]. 

The results show ZnCHA/alginate spheres are promising for bone tissue repair. Studies employing an animal grafting model observed the ZnCHA/alginate composites’ partial degradation, lack of large inflammatory infiltrates, and new bone formation after implantation [28,41]. The present findings show that the composite degradation process is important through ion desorption/absorption dynamics and particle release, relating it to the mineralization effect. Monitoring bone markers, F-Ost cells’ Runx-2 expression in ZnCHA reduced in 7 days, but mineralization nodule formation observed in 14 days reversed the effect. Further studies investigating mRNA expression for Runx-2, Col-I, OCN, and alkaline phosphatase may contribute to confirming the observed inductive effects on in vitro mineralization by ZnCHA/alginate composite spheres.

## 5. Conclusions

The results show that changes in the HA structure after the replacement of CO_3_^2−^ and Zn^2+^ caused a reduction in the crystallite size of the materials, as well as an increase in the surface area of the composites. The ZnCHA/alginate spheres gradually released concentrations of Zn and particles that, despite not causing cytotoxicity, altered the production profile of an osteogenic marker and resulted in increased in vitro mineralization. These results suggest ZnCHA/alginate composites may be promising materials as bone substitutes, making them strong candidates for future preclinical studies for tissue regeneration.

## Figures and Tables

**Table 1:** Composition of the evaluated biomaterials.

Tested Biomaterial	Composition	Chemical Formula
CHA	Carbonate apatite	Ca_10_(PO_4_)_6−x_(CO_3_)_x_(OH)_2−x_
ZnCHA	Carbonate apatite substituted with zinc	Ca_10−x_Zn_x_(PO_4_)_6−x_(CO_3_)_x_(OH)_2−x_

**Table 2:** Chemical analysis and parameters of crystallite extract from DRX the power samples.

	Chemical Analysis					Parameters of Crystallite			
	Ca%	*p*%	Water%	Zn%	Ca/P Ratio	Plan	2θ	D (nm)	Plans 002/300
CHA	38.4 ± 1.2	18 ± 0.5	2.4 ± 0.2	−	1.64	(002)	25.96	32.61	2.1 ± 0.3
						(300)	33.98	13.44	
ZnCHA	37.9 ± 1.1	18 ± 0.7	3.3 ± 03	3 ± 0.3	1.62	(002)	25.98	21.29	1.8 ± 0.7
						(300)	33.67	9.19	

D = crystallite size.

**Table 3:** Analysis of surface area, mean pore size, and pore volume of CHA and ZnCHA microspheres.

Samples	Specific Area (m^2^/g)	Mean Pore Diameter (nm)	Pore Volume (cm^3^/g)
CHA	136 ± 11.7	15 ± 0	0.5 ± 0.02
ZnCHA	187 ± 11	14 ± 0	0.6 ± 0.03

## Data Availability

The data presented in this study are available on request from the corresponding author. The data are not publicly available due to privacy reasons.
